# Mitochondrial Carrier Homolog 2 Functionally Co-operates With BH3 Interacting-Domain Death Agonist in Promoting Ca^2+^-Induced Neuronal Injury

**DOI:** 10.3389/fcell.2021.750100

**Published:** 2021-10-11

**Authors:** Beatrice D’Orsi, Natalia Niewidok, Heiko Düssmann, Jochen H. M. Prehn

**Affiliations:** ^1^Department of Physiology & Medical Physics, Centre for the Study of Neurological Disorders, Royal College of Surgeons in Ireland, Dublin, Ireland; ^2^Institute of Neuroscience, Italian National Research Council, Pisa, Italy

**Keywords:** excitotoxicity, calcium, Bcl-2 family, cortical neurons, permeability transition pore (mPTP), necrosis

## Abstract

The BH3 interacting-domain death agonist (BID) is a pro-apoptotic member of the Bcl-2 protein family. While proteolytic processing of BID links death receptor-induced apoptosis to the mitochondrial apoptosis pathway, we previously showed that full length BID also translocates to mitochondria during Ca^2+^-induced neuronal cell death. Moreover, mitochondrial carrier homolog 2 (MTCH2) was identified as a mitochondrial protein that interacts with BID during cell death. We started our studies by investigating the effect of *Mtch2* silencing in a well-established model of Ca^2+^-induced mitochondrial permeability transition pore opening in non-neuronal HCT116 cells. We found that silencing of *Mtch2* inhibited mitochondrial swelling and the associated decrease in mitochondrial energetics, suggesting a pro-death function for MTCH2 during Ca^2+^-induced injury. Next, we explored the role of BID and MTCH2 in mediating Ca^2+^-induced injury in primary cortical neurons triggered by prolonged activation of NMDA glutamate receptors. Analysis of intracellular Ca^2+^ transients, using time-lapse confocal microscopy, revealed that neurons lacking *Bid* showed markedly reduced Ca^2+^ levels during the NMDA excitation period. These Ca^2+^ transients were further decreased when *Mtch2* was also silenced. Collectively, our data suggest that BID and MTCH2 functionally interact to promote Ca^2+^-induced neuronal injury.

## Introduction

The BH3-only protein BID (BH3-interacting domain death agonist) is a pro-apoptotic member of the Bcl-2 family of proteins. BID is widely expressed in many tissues, including the adult brain (Krajewska et al., 2002). Once it is proteolytically activated by caspase-8 or other cytosolic proteases, its truncated form tBID is a potent inducer of the mitochondrial apoptosis pathway (Luo et al., 1998; Chen et al., 2001; Stoka et al., 2001; Mandic et al., 2002; Polster et al., 2005), integrating death receptor and proteases signaling with the mitochondrial apoptosis pathway (Wang et al., 1996; Engel et al., 2010, 2011), and facilitates mitochondrial cristae reorganization during apoptosis (Scorrano et al., 2002; Frezza et al., 2006; Cogliati et al., 2013). While the importance of tBID in the apoptotic pathway is well established, other functions of BID in the control of non-apoptotic cell death and mitochondrial bioenergetics have also emerged. Similarly to tBID, it has been shown that also full-length BID is an effective inducer of apoptosis in primary embryonic fibroblasts (Sarig et al., 2003) and translocates to mitochondria in a model of excitotoxic neuronal injury associated with Ca^2+^ overloading (Ward et al., 2006). This translocation occurred in the absence of cleavage, as detected with a BID-Förster resonance energy transfer (FRET) probe. We also showed that overexpression of a non-cleavable, full-length BID is sufficient to induce cell death in neurons (König et al., 2007). BID has also been proposed as a key player in the stroke- and trauma-induced neuronal injury where non-apoptotic cell death predominantly occurs (C. Culmsee and Plesnila, 2006). *bid*-deficient neurons were protected against oxygen/glucose deprivation-induced injury *in vitro* (Culmsee et al., 2005; Landshamer et al., 2008; Martin et al., 2016) and *bid*-deficient animals showed reduced injury middle cerebral artery occlusion or brain and traumatic brain injury (Plesnila et al., 2001; Franz et al., 2002; Yin et al., 2002; Bermpohl et al., 2006). Regarding the roles of BID in mitochondrial energetics, BID has been demonstrated to interact with the 33 kDa mitochondrial carrier homolog 2 (MTCH2) protein, which resides at the mitochondrial outer membrane, where it acts as a mitochondrial receptor for BID (Grinberg et al., 2005; Cogliati and Scorrano, 2010; Zaltsman et al., 2010; Katz et al., 2012). MTCH2 has been characterized as a regulator of mitochondrial metabolism, motility, and calcium buffering (Maryanovich et al., 2015; Buzaglo-Azriel et al., 2016; Ruggiero et al., 2017). Interestingly, loss of MTCH2 also protected hematopoietic stem cells from irradiation-induced injury (Maryanovich et al., 2015). However, the significance of BID and MTCH2 and their functional interaction in neurons has yet to be explored. In the present study, we investigated the interplay of these two proteins in the settings of Ca^2+^-induced neuronal injury.

## Materials and Methods

### Material

Fetal bovine serum, horse serum, minimal essential medium (MEM), B27 supplemented Neurobasal medium, tetramethylrhodamine methyl ester (TMRM), Fluo-4 AM (acetoxymethylester) came from Bio Sciences. All other chemicals, including NMDA, MK-801 came in analytical grade purity from Sigma-Aldrich.

### Cell Lines

*Bax/Bak* double-deficient control and MTCH2 knockdown HCT116 human colon cancer cell lines were maintained in RPMI 1640 medium supplemented with 10% fetal bovine serum, 100 μg/ml penicillin, 2 mM glutamine, and 100 μg/ml streptomycin. *Bax/Bak* double-deficient HCT116 cells were kindly provided by Dr. R.J. Youle (National Institute of Neurological Disorders and Stroke). The cells were cultured at 37°C in a humidified atmosphere of 5% CO_2_. For transfection, cells were seeded in six-well plates or wilco dishes for 24 h before transfection using Metafectene (Biontex) as per the manufacturer’s instructions.

#### Gene-Targeted Mice

The generation and genotyping of *bid*^–/–^ mice has previously been described (Kaufmann et al., 2007). The *bid*^–/–^ mice was generated on an inbred C57BL/6 background, using C57BL/6 derived ES cells. Wild type (WT) and *bid*-deficient mice were generated and maintained in house in the RCSI Biological Research Facility. All mouse strains were backcrossed for >12 generations on an inbred C57BL/6 background. DNA was extracted from tail snips using High Pure PCR Template Preparation Kit (Roche, Sussex, United Kingdom). Genotyping was performed using three specific primers as follows: 5′GGTCTGTGTGGAGAGCAAAC3′ (common), 5′TCAGGTGCCAGTGGAGATGAACTC3′ (wild type allele-specific) and 5′GAGTCATACTTACTTCCTCCGAC3′ (mutant allele-specific) for *bid* (D’Orsi et al., 2012). All animal work was performed with ethics approval and under licenses granted by the Health Products Regulatory Authority (HPRA, Ireland) in accordance with European Communities Council Directive (86/609/EEC) and procedures reviewed and approved by the RCSI Research Ethics Committee.

### Preparation of Mouse Neocortical Neurons

Primary cultures of cortical neurons were prepared at embryonic gestation day 16–18 (E16–E18) (D’Orsi et al., 2012, 2015; D’Orsi et al., 2016). To isolate cortical neurons, hysterectomies of the uterus of pregnant female mice were carried out after euthanizing mice by cervical dislocation. The cerebral cortices were isolated from each embryo and pooled in a dissection medium on ice [PBS with 0.25% glucose, 0.3% bovine serum albumine (BSA)]. Tissue was incubated with 0.25% trypsin-EDTA at 37°C for 15 min. After incubation, trypsinization was stopped by the addition of fresh plating medium (minimal essential medium containing 5% fetal bovine serum, 5% horse serum, 100 U/ml penicillin/streptomycin, 0.5 mM L-glutamine and 0.6% D-glucose). Neurons were then dissociated by gentle pipetting and after centrifugation (1,500 rpm, 3 min), medium containing trypsin was aspirated. Neocortical neurons were resuspended in plating medium, plated at 2 × 10^5^ cells per cm^2^ on poly-D-lysine-coated plates (final concentration of 5 μg/ml), and then incubated at 37°C and 5% CO_2_. The plating medium was exchanged with 50% feeding medium (Neurobasal containing 100 U/ml of penicillin/streptomycin, 2% B27 and 0.5 mM L-glutamine) and 50% plating medium with additional mitotic inhibitor cytosine arabinofuranoside (600 nM). Two days later, medium was again exchanged with complete feeding medium. All experiments were performed on day 8–11 *in vitro* (DIV).

### Time-Lapse Live Cell Imaging

#### Intracellular Calcium Measurements Using Fluo4 AM

Primary neocortical neurons on Willco dishes (Willco Wells B.V.) were co-loaded with the calcium dye, Fluo-4 AM (3 μM), and the membrane-permeant cationic fluorescent probe, TMRM (20 nM) for 30 min at 37°C (in the dark) in experimental buffer containing (in mM): 120 NaCl, 3.5 KCl, 0.4 KH_2_PO_4_, 20 HEPES, 5 NaHCO_3_, 1.2 Na_2_SO_4_, 1.2 CaCl_2_, and 15 glucose; pH 7.4. Cells were washed and bathed in 2 ml of experimental buffer containing 20 nM TMRM and a thin layer of mineral oil was added to prevent evaporation. Neurons were placed on the stage of a LSM 510 Meta confocal microscope equipped with a 63 × 1.4 NA oil immersion objective and a thermostatically regulated chamber (Carl Zeiss Jena). Following 30 min equilibration time, neurons were exposed to 300 μM NMDA plus 10 μM glycine for 1 h; MK-801 (5 μM) was added to terminate NMDA receptor activation as required (D’Orsi et al., 2012). TMRM was excited at 543 nm and the emission collected with a 560 nm long pass filter. Fluo-4 was excited at 488 nm and the emission was collected through a 505–550 nm barrier filter. Images were captured every 30 s during NMDA excitation and every 5 min during the rest of the experiments. For the *Mtch2* gene silencing single cell experiments, neurons were co-transfected with a vector silencing *Mtch2* (SHH346164; Creative Biogene) and a plasmid expressing enhanced CFP (ECFP-C1, BD Bioscience Clontech), or for control neurons, transfected with the CFP plasmid only. Neocortical neurons were transfected at DIV 6 using Lipofectamine 2000 (Invitrogen). Two days after transfection, neurons were co-loaded with Fluo-4 AM (3 μM) and TMRM (20 nM) in experimental buffer, and placed on the stage of a LSM 710 confocal microscope equipped with a 63 × 1.4 NA oil immersion objective and a thermostatically regulated chamber set at 37°C (Carl Zeiss). Following a baseline equilibration time, NMDA dissolved in experimental buffer was added to the medium. TMRM was excited at 561 nm, and the emission was collected in the range of 575–735 nm. Fluo-4 was excited at 488 nm and the emission was collected in the range of a 505–550 nm. All microscope settings including laser intensity and scan time were kept constant for the whole set of experiments. Control experiments were carried out and showed that photo toxicity had a negligible impact. All images were processed and analyzed using MetaMorph Software version 7.5 (Universal Imaging Co.), and the data presented were normalized to the baseline.

#### Single-Cell Mitochondrial ATP Measurements With mitoATeam

*Bax/Bak* double-deficient HCT116 control and MTCH2 Kd cells, transfected with the FRET-based mitochondrial ATP indicator, mitoATeam, and loaded with TMRM (30 nM) in Krebs buffer containing (in mM): 140 NaCl; 5.9 KCl; 1.2 MgCl_2_; 15 HEPES; 2.5 CaCl_2_ and 10 glucose; pH 7.4, were placed on the stage of a LSM 710 confocal microscope equipped with a 63 × 1.4 NA oil-immersion objective and thermostatically regulated chamber set at 37°C (Carl Zeiss). After a 10 min equilibration time, cells were exposed to sequential addition of 1 μM Ionomycin for 10 min, 10 μM Ionomycin for 10 min and 10 μM FCCP to cause disruption of ATP synthesis. TMRM was excited at 561 nm, and the emission was collected in the range of 575–735 nm. CFP was excited at 405 nm, and emission was collected at 445–505 (CFP) and 505–555 nm (FRET). Yellow fluorescent protein (YFP) was excited directly using the 488 nm line of the Argon Laser and detected in the same range used for FRET. Images were captured every 1 min throughout these experiments.

#### Imaging of Mitochondrial Permeability Transition Pore Opening

Mitochondrial permeability transition pore (mPTP) opening was examined by calcein release from mitochondria in *Bax/Bak* double-deficient HCT116 control and MTCH2 Kd cells. Cells, plated on small Willco dishes, were first loaded with calcein-AM (1 μM) for 30 min in Krebs buffer. After that, CoCl_2_ (1 mM) was added for 10 min to quench cytosol calcein and cells were then bathed in 150 μl of Krebs solution containing TMRM (30 nM) and covered by a thin layer of mineral oil. The cells were placed on the stage of a LSM 710 confocal microscope equipped with a 63 × 1.4 NA oil-immersion objective and thermostatically regulated chamber set at 37°C (Carl Zeiss). Calcein AM and cobalt enter the cell, where the AM groups are cleaved from calcein via non-specific esterase activity in the cytosol and mitochondria. Cobalt cannot enter healthy mitochondria and quenches the cytosolic calcein signal. Upon opening of the mPTP, cobalt enters through the pore and subsequently quenches the mitochondrial calcein fluorescence. Calcein was excited at 488 nm and emission was collected in the range of 505–550 nm. TMRM was excited at 561 nm, and the emission was collected in the range of 575–735 nm. mPTP opening was indicated by a reduction in mitochondrial calcein signal, measured every minute, and expressed as standard deviation of calcein fluorescence.

### Isolation of Functional Mitochondria and Measurement of Swelling

Mitochondria were isolated from *Bax/Bak* double-deficient human HCT116 colon cancer control and MTCH2 Kd cells following the methods of Frezza et al. (2007). Protein was measured using Bradford reagent (Sigma). Mitochondrial pellets were resuspended in experimental buffer (125 mM KCl, 20 mM Hepes, 2 mM KH_2_PO_4_, 1 μM EGTA, 4 mM MgCl_2_, 3 mM ATP, 5 mM malate and 5 mM glutamate) (Chinopoulos et al., 2003) at a final concentration of 1 mg/ml and 100 μl was added to a 96-well flat bottomed transparent plate. Absorbance at 540 nm was detected in each well at 30 s intervals in a BioTek Synergy HT microplate reader and CaCl_2_ (100 μmol/l) was used. Alamethicin (80 μg/ml) was then added to induce maximal swelling. All experiments were carried out on at least three separate preparations to ensure reproducibility of results.

### Western Blotting

Preparation of cell lysates from HCT116 cells was carried out as previously described (D’Orsi et al., 2015). The resulting blots were probed with either: a rabbit monoclonal MTCH2 antibody (ab7977; Abcam) 1:250; a mouse monoclonal β-actin antibody (clone DM 1A; Sigma) diluted 1:5,000. Horseradish peroxidase conjugated secondary antibodies diluted 1:10,000 (Pierce) were detected using Immobilon Western Chemiluminescent HRP Substrate (Millipore) and imaged using a FujiFilm LAS-3000 imaging system (Fuji).

### Statistical Analysis

Data are given as means ± SEM (standard errors of the means). Data were analyzed using one-way analysis of variance (ANOVA) followed by Tukey’s *post hoc* test or Student’s *t*-test for two-group comparison. *P* values <0.05 were considered to be statistically significant. When significant, exact *p* values were stated in the figure legends.

## Results

### *Mtch2* Gene Silencing Delays Ca^2+^-Induced Mitochondrial Dysfunction in Non-neuronal Cells

**FIGURE 1:**
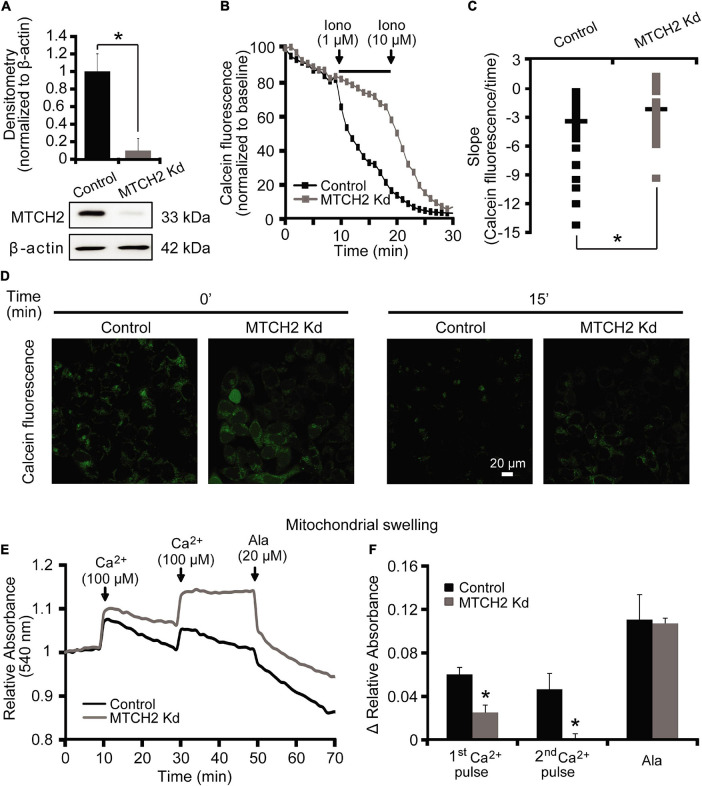
MTCH2 modulates mPTP opening in HCT116 cells. **(A)** Bax/Bak double-deficient HCT116 cells were infected with a lentivirus shRNA vector against Mtch2 or scramble shRNA. Reduced protein levels of MTCH2 after shRNA expression compared to the scramble were assessed by quantitative Western blotting 72 h post-transduction. Probing for β-actin served as loading control. Experiments were repeated three times with similar results. Densitometric data are normalized to β-actin. Means ± SEM are shown. *p ≤ 0.05 compared to Bax/Bak double-deficient control HCT116 cells (ANOVA, *post hoc* Tukey). **(B–D)** Cobalt-quenching assay was performed in Bax/Bak double-deficient MTCH2 kd and control HCT116 cells. Mitochondrial calcein leakage was analyzed after sequential addition of ionomycin (1 and 10 μM) and representative traces are shown **(B)**. Quantification of the slope (the change in FRET/CFP ratio over time in minutes) between 10 and 20 min time point (corresponding to the two additions of ionomycin) is illustrated **(C)**. Representative images of calcein-CoCl_2_ staining of Bax/Bak double-deficient MTCH2 kd and control HCT116 cells before (*t* = 0 min) and after the first addition of ionomycin (*t* = 15 min) are shown **(D)**. Scale bar = 20 μM. A minimum of 40 cells from at least *n* = 3 independent experiments were analyzed per cell type/condition. *p ≤ 0.05 compared to Bax/Bak double-deficient control HCT116 cells (ANOVA, *post hoc* Tukey). **(E,F)** Mitochondria isolated from Bax/Bak double-deficient MTCH2 kd and control HCT116 cells as described in the section “Materials and Methods,” were pre-incubated at 30°C in experimental buffer in a 96-well plate at a final volume of 100 μl per well. Absorbance at 540 nm was detected at 30 s intervals and CaCl_2_ (100 μM) was added where shown **(E)**. Alamethicin (20 μM) was added to induce maximal swelling. Results represent average traces from *n* = 3 experiments and Δ relative absorbance was calculated at the indicated points **(F)**. *p ≤ 0.05 compared to Bax/Bak double-deficient control HCT116.

We started our experiments by characterizing a possible role for MTCH2 in the control of Ca^2+^-induced mitochondrial dysfunction and cell death in a model of Ca^2+^-induced mPTP opening. Neurons are largely resistant to Ca^2+^-induced mPTP opening (Brustovetsky and Dubinsky, 2000; Chinopoulos et al., 2003). We therefore set out our investigations in a model of Ca^2+^-induced injury in human HCT116 colon cancer cells. In order to exclude the possibility that apoptosis-induced MOMP contributed to any effects observed, Ca^2+^-mediated mitochondrial injury was induced in *Bax/Bak* double-deficient human HCT116 colon cancer cells silenced for *Mtch2*. Using Western blotting, we first verified the efficiency of the silencing of the *Mtch2* gene in these cells. Reduced levels of MTCH2 were observed in the *Bax/Bak* double-deficient HCT116 MTCH2 Kd cells, obtained 72 h post lentiviral transduction with a shRNA construct when compared to cultures infected with a scramble control vector (Figure 1A).

To examine a requirement for MTCH2 in mPTP opening in intact cells, we performed a calcein/Co^2+^-quenching assay (Sun et al., 2014) in the *Bax/Bak* double-deficient HCT116 control and MTCH2 Kd cells. The calcein/Co^2+^ assay is based on the concept that mitochondrial calcein fluorescence is quenched by Co^2+^ after the irreversible opening of the mPTP. Cells were exposed to sequential addition of the Ca^2+^ ionophore ionomycin (1 and 10 μM). When 1 μM ionomycin was added to *Bax/Bak* double-deficient HCT116 MTCH2 Kd cells, calcein fluorescent signals remained within mitochondria (Figures 1B,D, gray), whereas significant calcein leakage from mitochondria was seen in *Bax/Bak* double-deficient HCT116 control cells (Figures 1B,D, black). Addition of 10 μM ionomycin resulted in a decrease in calcein fluorescence also in the *Bax/Bak* double-deficient HCT116 MTCH2 Kd cells (Figure 1B), suggesting that the knockdown delayed rather than prevented mPTP opening. Detailed analysis of individual calcein fluorescence traces showed a significant reduction in the slope of calcein fluorescent signal after 1 μM ionomycin in the *Bax/Bak* double-deficient HCT116 MTCH2 Kd cells compared to their control (Figure 1C).

Next, control and MTCH2 Kd mitochondria were isolated from *Bax/Bak* double-deficient HCT116 cells. Ca^2+^-induced mitochondrial swelling was detected by a rapid loss of absorbance at 540 nm in cells exposed to serial addition of 100 μM Ca^2+^ and 20 μM of Alamethicin. Alamethicin is a membrane-channel-forming peptide that induces mitochondrial swelling similar to mPTP (Andreyev et al., 1998; Andreyev and Fiskum, 1999), which was used in this model as a positive control to produce maximal swelling of mitochondria. Mitochondrial swelling in response to Ca^2+^ addition was more pronounced in mitochondria isolated from *Bax/Bak* double-deficient HCT116 control cells compared to those where *Mtch2* was silenced (Figures 1E,F). Altogether, these data suggest that MTCH2 was required for effective ionomycin- and Ca^2+^-induced mPTP opening.

**FIGURE 2:**
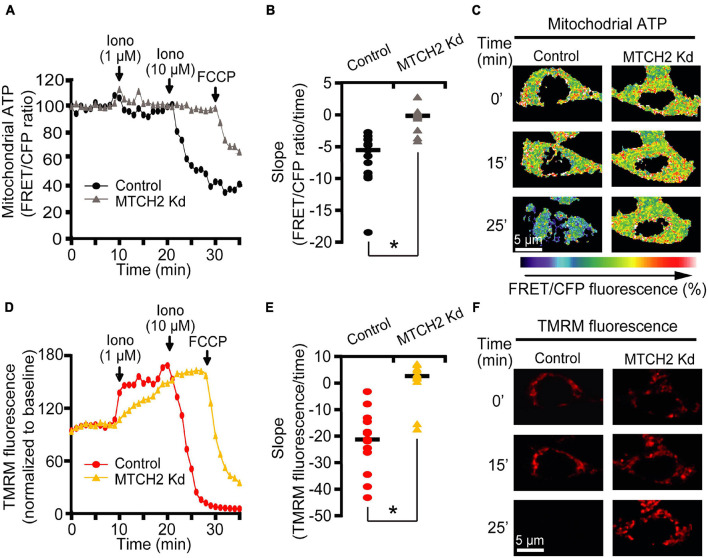
MTCH2 influences cellular mitochondrial bioenergetics in HCT116 cells. **(A–F)** *Bax/Bak* double-deficient MTCH2 kd and control HCT116 cells were separately transfected with the mitochondria ATP-sensitive (mitoATeam) FRET probe, loaded with 30 μM TMRM as a Δψ_m_ indicator (non-quenched mode), and mounted on the stage of LSM 710 confocal microscope. Fluorescent measurements were recorded for TMRM, FRET, CFP, and YFP by time-lapse confocal microscope. FRET probe imaging data are expressed as a ratio of FRET/CFP. Cell were exposed to sequential addition of 1 and 10 μM ionomycin, and FCCP (1 μM) at the indicated time points. Mitochondrial ATP **(A)** and Δψ_m_ **(D)** were analyzed after drugs addition and representative traces are shown. Quantification of the slope of mitochondrial ATP [the change in FRET/CFP ratio over time in minutes; **(B)**] and Δψ_m_ [the change in TMRM fluorescence over time in minutes; **(E)**] between 20 min time point (corresponding to the 10 μM ionomycin treatment) and the end of experiment is illustrated. Representative images of mitoATeam-transfected **(C)** and TMRM-loaded **(F)** *Bax/Bak* double-deficient MTCH2 kd and control HCT116 cells before (*t* = 0 min) and following the additions of ionomycin (*t* = 15 min and *t* = 25 min, respectively) are shown. Scale bar = 5 μM. A minimum of 15 cells from at least *n* = 3 independent experiments were analyzed per cell type/condition. *p ≤ 0.05 compared to *Bax/Bak* double-deficient control HCT116 cells (ANOVA, *post hoc* Tukey).

To assess whether MTCH2 knockdown may affect mitochondrial bioenergetics in this model, we also monitored mitochondrial ATP levels at single cell resolution, using an ATP-sensitive FRET probe targeted to the mitochondria, mitoATeam (Imamura et al., 2009). In parallel, we measured mitochondrial membrane potential (Δψ_m_) by employing the membrane-permeant cationic fluorescent probe, TMRM. Again, experiments were conducted in *Bax/Bak* double-deficient HCT116 control and MTCH2 Kd cells by time-lapse confocal microscopy. Individual single cell analysis revealed similar mitochondrial ATP dynamics between control and MTCH2 kd cells following the first addition of 1 μM ionomycin, while 10 μM ionomycin triggered a rapid mitochondrial ATP reduction in the control cells but not in those where *Mtch2* was silenced, as shown by the analysis of the slope of the FRET/CFP ratio mitochondrial signal (Figures 2A–C). Of note, *Bax/Bak* double-deficient HCT116 MTCH2 Kd cells displayed partial mitochondrial ATP depletion only when subjected to the mitochondrial uncoupler FCCP (Figures 2A–C). Similarly, analysis of Δψ_m_ displayed that *Mtch2* gene silencing produced a significant delay in the onset of Δψ_m_ depolarization. *Bax/Bak* double-deficient HCT116 MTCH2 Kd cells showed Δψ_m_ loss only following FCCP exposure, as observed also by analysis of the slope of the TMRM fluorescence, while controls exhibited a complete Δψ_m_ depolarization immediately after 10 μM ionomycin treatment (Figures 2D–F), suggesting that MTCH2 decreased cellular bioenergetic capacity. Collectively, these experiments provided evidence for a role of the BID receptor MTCH2 in Ca^2+^-induced mitochondrial dysfunction and cell death.

### NMDA-Induced Ca^2+^ Overloading Is Not Significantly Reduced by *Mtch2* Gene Silencing in Primary Cortical Neurons, but Sensitive to *Bid* Gene Deletion

**FIGURE 3:**
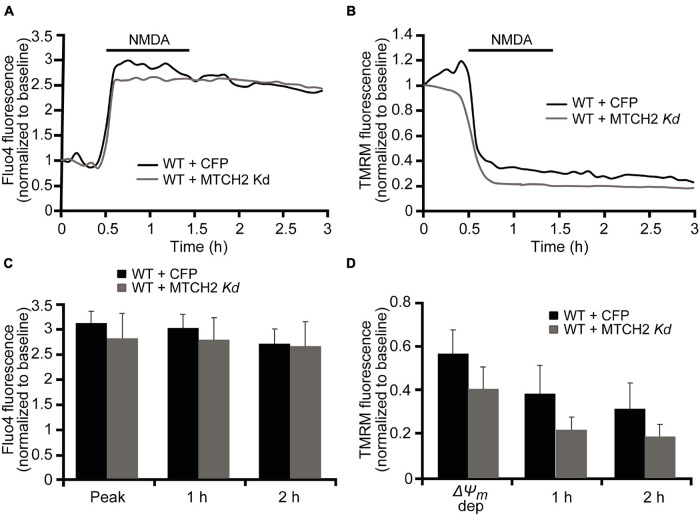
*Mtch2* gene silencing does not diminish NMDA-induced Ca^2+^ in WT cortical neurons. WT cortical neurons, transfected with an shRNA plasmid against Mtch2 or a control empty vector (ECFP) for 36 h and co-loaded with TMRM (20 nM) and Fluo-4 AM (3 μM), were exposed to NMDA (300 μM/60 min NMDA) and monitored by confocal microscopy (LSM 710). **(A,B)** Representative Fluo4-AM **(A)** and TMRM **(B)** traces measuring alterations in intracellular Ca^2+^ influx and Δψ_m_, respectively, at point of stimulation (300 μM/60 min NMDA) and following the initial excitotoxic stimulus. **(C)** Analysis of the relationship Fluo-4 fluorescence at the indicated time points compared to peak Fluo-4 AM fluorescence at NMDA exposure. WT cortical neurons transfected with Mtch-2 kd plasmid: *n* = 18 neurons from *n* = 5 separate cultures. Empty vector: *n* = 25 neurons from *n* = 5 separate cultures. Data are shown as means ± SEM (*p* ≥ 0.05; ANOVA, *post hoc* Tukey’s test). **(D)** Average of TMRM fluorescence in WT cortical neurons transfected with Mtch-2 kd plasmid (*n* = 18; from *n* = 5 separate cultures) or control vector (*n* = 25; from *n* = 5 separate cultures) during and after NMDA excitation. Means ± SEM are shown (*p* ≥ 0.05; ANOVA, *post hoc* Tukey’s test).

In order to investigate whether MTCH2 and the BH3-only protein BID were involved in Ca^2+^-induced mitochondrial dysfunction and cell death in neurons, we employed a more physiological, established model of NMDA-mediated Ca^2+^ excitotoxicity that preferentially produced excitotoxic necrosis in cortical neurons. We previously demonstrated that duration and severity of the excitotoxic stimulus determines different neuronal outcome and, that exposure of mature cortical neurons to 300 μM NMDA for 60 min induces an immediate neuronal death within 1 h (Weisová et al., 2011; D’Orsi et al., 2012). As we have seen significant effects of MTCH2 in the model of Ca^2+^ -induced injury in HCT116 cells, we first focused on the role of *Mtch2* gene silencing. Wild-type (WT) cortical neurons were transfected with the plasmid silencing *Mtch2* and co-transfected with a CFP-expressing vector (WT + MTCH2 kd) or only transfected with an empty vector (WT + CFP), and then exposed to 300 μM NMDA for 60 min. Analysis of WT-*Mtch2* silenced neurons revealed no significant changes in cytosolic Ca^2+^ transients compared to their control neurons during NMDA-induced excitotoxic injury (Figures 3A,C). Quantification of the individual TMRM responses also showed similar levels of TMRM fluorescence compared to the control transfected WT neurons (Figures 3B,D). These findings suggested that in neurons MTCH2 may need to cooperate with other factors to regulate Ca^2+^-induced injury.

**FIGURE 4:**
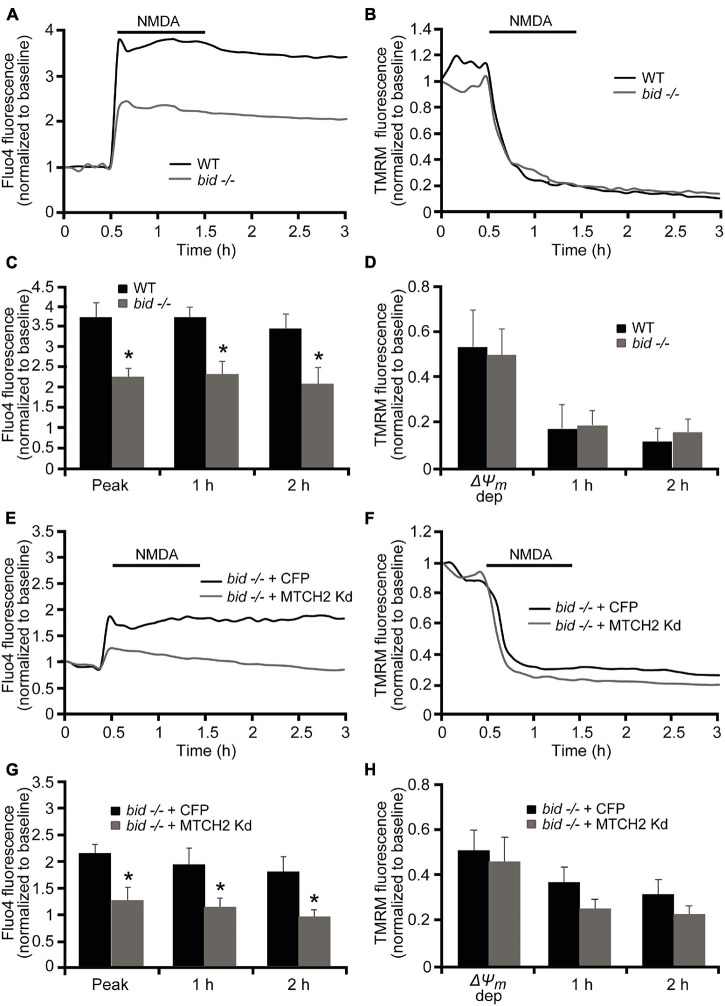
*Mtch2* gene silencing attenuates NMDA-induced Ca^2+^ overloading in *bid*-deficient cortical neurons. **(A–D)** WT and *bid-*deficient cortical neurons, cultured separately on Willco dishes, were preloaded with TMRM (20 nM) and Fluo-4 AM (3 μM) for 30 min at 37°C before being monitored by a confocal microscope (LSM 510Meta). Neurons exposed to 300 μM NMDA for 60 min were monitored in single-cell imaging for alterations in TMRM fluorescence and intracellular Ca^2+^ over a 3 h period. Representative traces of NMDA-treated WT and *bid*-deficient cortical neurons depicting the extent of peak Ca^2+^ influx at point of stimulation and ICD **(A)**, and TMRM fluorescence loss **(B)** after the excitotoxic stilmulus (300 μM/60 min NMDA) are shown. Analysis of the Fluo-4 fluorescence **(C)** and average of TMRM fluorescence **(D)** at the indicated time points during and after NMDA exposure (300 μM/60 min) was performed in WT (*n* = 83) and *bid*-deficient (*n* = 43) neurons. Data are means ± SEM from at least *n* = 3 independent experiments for each genotype. *p ≤ 0.05 compared to NMDA-treated WT controls (ANOVA, *post hoc* Tukey). **(E–H)** *bid*-deficient neurons, transfected with an shRNA plasmid against Mtch2 or a control empty vector (ECFP) for 36 h and co-loaded with TMRM (20 nM) and Fluo-4 AM (3 μM), were exposed to NMDA (300 μM/60 min NMDA) and monitored by confocal microscopy (LSM 710). Representative Fluo4-AM **(E)** and TMRM **(F)** traces measuring alterations in intracellular Ca^2+^ influx and TMRM fluorescence, respectively, at point of stimulation (300 μM/60 min NMDA) and following the initial excitotoxic stimulus. Analysis of the relationship Fluo-4 fluorescence at the indicated time points compared to peak Fluo-4 AM fluorescence at NMDA exposure **(G)**. *bid*-deficient cortical neurons transfected with Mtch-2 kd plasmid: *n* = 20 neurons from *n* = 5 separate cultures. Empty vector: *n* = 28 neurons from *n* = 5 separate cultures. Data are shown as means ± SEM. *p ≤ 0.05 compared to NMDA-treated *bid*-deficient controls (ANOVA, *post hoc* Tukey). Average of TMRM fluorescence in *bid*-deficient cortical neurons transfected with Mtch-2 kd plasmid (*n* = 20; from *n* = 5 separate cultures) or control vector (*n* = 28; from *n* = 5 separate cultures) during and after NMDA excitation **(H)**. Means ± SEM are shown (*p* ≥ 0.05; ANOVA, *post hoc* Tukey’s test).

Next, primary mouse cortical neurons were derived from *bid*-deficient mice and compared to WT mice. Quantification of individual Ca^2+^ responses, using Fluo-4 AM, following exposure to 300 μM NMDA for 60 min demonstrated that the majority of WT neurons failed to recover their NMDA-induced intracellular Ca^2+^ increase and underwent immediate Ca^2+^ deregulation (ICD), while Ca^2+^ overloading in *bid*-deficient neurons was significantly reduced during and post NMDA excitation (Figures 4A,C). Loss of Bid had no significant effect on TMRM fluorescence changes during and after the NMDA exposure (Figures 4B,D).

### *Mtch2* Silencing Further Decreases NMDA-Induced Immediate Neuronal Calcium Dysregulation in *bid*-Deficient Neurons

Next, we investigated whether BID functionally interacts with MTCH2 in the control of NMDA-induced excitotoxic Ca^2+^ overloading. *bid*-deficient cortical neurons were first transfected with a plasmid silencing *Mtch2* and co-transfected with a CFP-expressing vector (*bid^–/–^* + MTCH2 kd) or only transfected with an empty vector (*bid^–/–^* + CFP), in analogy to the experiments described in Figure 3, and then exposed to 300 μM NMDA for 60 min. While WT-*Mtch2* silenced neurons revealed no significant changes in cytosolic Ca^2+^ transients compared to their control neurons, as previously shown in Figure 3, *bid*-deficient neurons silenced of *Mtch2* exhibited significantly lower cytosolic Ca^2+^ levels in response to the NMDA challenge compared to their control, both at the point of NMDA exposure and at later time points (Figures 4E,G). However, quantification of the individual TMRM responses showed no significant alterations in the Δψ*_m_* in neurons where *Mtch2* gene was silenced during and after NMDA exposure (Figures 4F,H).

## Discussion

In this study, we set out to explore the role of BID in neuronal excitotoxicity and how it relates with its interactor MTCH2, specifically in the setting Ca^2+^-induced neuronal death. First, we demonstrated that, in a non-neuronal system, such as the human HCT116 colon cancer cells, MTCH2 is essential for Ca^2+^-induced mPTP opening and collapse of mitochondrial energetics. Conversely, in a neuronal model, MTCH2 did not play a crucial role in NMDA-induced Ca^2+^ overloading on its own, but it needed to functionally co-operate with BID to explicate this function.

In the recent years, several studies have been focused in analyzing the effects of the loss of MTCH2 in various cell models and systems, mainly showing that Mtch2 deficiency results in alterations in mitochondrial functions and metabolism, calcium dynamics and embryonic development. In detail, Mtch2 deletion caused embryonic lethality before E7.5 stage (Zaltsman et al., 2010) and impaired mitochondrial architecture, in particular, producing increased mitochondrial fragmentation, decreased elongation and fusion rate in both murine embryonic fibroblasts and embryonic stem cells, suggesting that MTCH2 is a direct regulator of mitochondrial fusion (Bahat et al., 2018). Moreover, another team of researchers showed that loss of MTCH2 results in changes in mitochondria morphology and increased mitochondria functions, including oxidative phosphorylation, mitochondrial size, and ATP, NADH, and ROS levels in hematopoietic stem cell (Maryanovich et al., 2015), greater mitochondrial mass and metabolism as well as the whole-body energy homeostasis in mice in vivo (Buzaglo-Azriel et al., 2016), and reduced mitochondrial motility and calcium handling in hippocampal neurons (Ruggiero et al., 2017). Our study supports these previously reported activities, but also gives new important insights in MTCH2 role in Ca^2+^-induced injury. In fact, our data obtained from single-cell imaging experiments suggested that *Mtch2* silencing delayed Ca^2+^-induced mPTP opening and decreased mitochondrial swelling and bioenergetics in Bax/Bak double-deficient colon cancer cells, indicating that the MTCH2 is required, and *per se* sufficient, for the preservation of mitochondrial energetics (Figures 1, 2). As neurons are mainly resistant to Ca^2+^-induced mPTP opening (Brustovetsky and Dubinsky, 2000; Chinopoulos et al., 2003), to study the role of MTCH2 in a neuronal system, we moved toward a well-established model of NMDA-mediated Ca^2+^ excitotoxicity that mostly results in excitotoxic necrosis in cortical neurons (D’Orsi et al., 2012). Intriguingly, *Mtch2* silencing failed to exert significant alterations in calcium dynamics in WT cultured cortical neurons exposed to NMDA (Figure 3). This lead us to the question whether MTCH2 required other factors to regulate Ca^2+^-induced injury? Indeed, MTCH2, other than being a critical regulator of mitochondrial energetics, has been demonstrated to also interacts with tBID facilitating its recruitment to the mitochondria to regulate apoptosis (Grinberg et al., 2005; Cogliati and Scorrano, 2010; Zaltsman et al., 2010).

To date, what is well known about BID is that, in certain cell types, this BH3-only member of the Bcl-2 family integrates death receptor activation to the mitochondrial apoptosis pathway (Li et al., 1998; Luo et al., 1998; Gross et al., 1999). BID is cleaved by caspase 8 to its truncated form tBID, which subsequently leads to the release of cytochrome c by causing oligomerization of BAX and BAK that results in MOMP (Hardwick and Youle, 2009) and by inducing OPA1-dependent cristae remodeling (Frezza et al., 2006). BID has also been suggested as key mediator role of cell death in several neurodegeneration models, in particular contributing to post-ischemic neuronal cell death (C. Culmsee and Plesnila, 2006), but here its functional involvement may be multifaceted and injury-dependent. For example, we showed that not only tBID, but also full-length BID translocates to mitochondria inducing excitotoxic neuronal death (Ward et al., 2006; König et al., 2007). In line with recent findings from our group (Martin et al., 2016), BID has been previously implicated in oxygen/glucose deprivation (OGD)-induced neuronal death in neurons, through the activation of caspase 8 after focal cerebral ischemia (Plesnila et al., 2001; Le et al., 2002). BID inhibition also prevented nuclear translocation of the pro-apoptotic factor AIF in models of hypoxia–hypoglycemia-induced neuronal cell death (Culmsee et al., 2005), although the role of caspase 8 in AIF release from mitochondria was not elucidated in this study, and a role for PARP activation was proposed. Polster et al. (2005) suggested in isolated mitochondria studies that the activation of caspase 8 by BID was not sufficient to induce release of pro-apoptotic AIF from mitochondria, but that BID cleavage and the release of AIF was calpain-dependent (Polster et al., 2005).

Here, our data demonstrate that BID may play a critical role in contributing to excitotoxic injury under conditions that are associated with immediate Ca^2+^ deregulation (ICD; Figure 4), and that it here functionally interacts with MTCH2. *Bid* gene deletion *per se* produced decreased levels of Ca^2+^ peak responses and reduced immediate Ca^2+^ deregulation, denoting that BID may have an effect on neuronal Ca^2+^ buffering and that Ca^2+^ deregulation and cell death may be coupled. While BID may trigger MOM permeabilization through activation of pro-apoptotic proteins such as BAX (Kuwana et al., 2002), it has also been involved in disturbing mitochondrial dynamics by disrupting the assembly of OPA1 oligomers, thereby causing cristae remodeling and mitochondrial fragmentation (Kroemer et al., 2007). Of note, when *bid*-deficient neurons were knocked down of MTCH2, the NMDA-induced Ca^2+^ transients were additionally reduced both at the point of NMDA stimulation and at later time points post-NMDA exposure (Figure 4), a result that was not observed in WT neurons where *Mtch2* was silenced in the same condition (Figure 3). This indicates a mechanistic interplay between BID and MTCH2 in the Ca^2+^-induced cell death processes in neurons. Our data also showed that, while the double concomitant deficiency of *Bid* and *Mtch2* had an obvious effect on NMDA-mediated Ca^2+^ dynamics, this was not significantly detected in terms of alterations in TMRM fluorescence levels, used to quantify mitochondrial membrane potential (Figures 3, 4). Nevertheless, it should be noted that in models of prolonged NMDA receptor overactivation, plasma membranes also depolarize (Ward et al., 2007). Of note, TMRM fluorescence intensity is sensitive to changes in both mitochondrial and plasma membrane potential (Nicholls and Ward, 2000; Ward et al., 2007), limiting the interpretations of TMRM traces in the setting of prolonged NMDA receptor activation. Additional mechanisms of Ca^2+^ homeostasis regulation by BID may also contribute; Bcl-2 family proteins have been demonstrated to be involved in the regulation of the interaction between mitochondria and ER by influencing ER calcium stores and signaling (Scorrano et al., 2003; Oakes et al., 2005; Rong et al., 2009; D’Orsi et al., 2015, 2017). However, these suggested potential mechanisms would need further investigation. Zaltsman et al. (2010) suggested that mitochondrial accumulation of tBID relies on MTCH2 that serves as a receptor-like protein for tBID (Zaltsman et al., 2010). Recently, it has been demonstrated that the receptor function of MTCH2 in binding tBID requires interaction with a Bax-binding protein enriched at the MOM, MOAP-1, in a model of Fas-induced apoptosis in liver. This interaction allows for a MTCH2 conformation change that facilitates tBID-MTCH2 binding, thereby leading to recruitment of tBID to mitochondria (Tan et al., 2016).

In conclusion, our results demonstrate that, in neurons, the BH3-only protein BID functionally acts together with its mitochondrial receptor MTCH2 in regulating NMDA-induced Ca^2+^ overloading.

## Data Availability Statement

The raw data supporting the conclusions of this article will be made available by the authors, without undue reservation.

## Ethics Statement

The animal study was reviewed and approved by Health Products Regulatory 93 Authority (HPRA, Ireland) in accordance with European Communities Council Directive 94 (86/609/EEC) and procedures reviewed and approved by the RCSI Research Ethics Committee. Written informed consent was obtained from the owners for the participation of their animals in this study.

## Author Contributions

BD’O, NN, HD, and JP designed the study. BD’O, NN, and HD designed and performed the experiments and analyzed and interpreted data. BD’O and JP wrote the manuscript. JP acquired funding. All authors contributed to the article and approved the submitted version.

## Conflict of Interest

The authors declare that the research was conducted in the absence of any commercial or financial relationships that could be construed as a potential conflict of interest.

## Publisher’s Note

All claims expressed in this article are solely those of the authors and do not necessarily represent those of their affiliated organizations, or those of the publisher, the editors and the reviewers. Any product that may be evaluated in this article, or claim that may be made by its manufacturer, is not guaranteed or endorsed by the publisher.
